# Supplementary material for: Quantifying political influence on COVID-19 fatality in Brazil
Source: PLoS One. 2022 Jul 12;17(7):e0264293. doi: 10.1371/journal.pone.0264293 (PMC9275831; doi:10.1371/journal.pone.0264293)
Supplement: S1 File — (PDF) [file pone.0264293.s001.pdf]

Data Source The data was retrieved from TSE using the website "Gazeta do Povo" and compiled at: `https://especiais.gazetadopovo.com.br/eleicoes/2018/resultados/mapa-eleitoral-de-presidente-por-municipios-2turno`

COVID19 Deaths confirmed by state All cases reported here were confirmed by the health departments of each state, and also obtained by the official platform of the Ministry of Health. All data is available in CSV format on Github: `https://github.com/wcota/covid19br` [21]. The fatality data and votes were related using the Pearson Coefficient and the code is avaliable at [22].

Pearson correlation coefficient The Pearson correlation coefficient is named for Karl Pearson and gives us the strength of the linear relationship between two data samples. It is calculated as the covariance of the two variables divided by the product of the standard deviation of each data sample. It is the normalization of the covariance between the two variables to give an interpretable score:

$$R_{X,Y} = \frac{cov(X,Y)}{\sigma_X \sigma_Y}\,, \tag{1}$$

where $cov(X,Y)$ is the covariance between X and Y,

$$cov(X,Y) = \sum_{i=1}^{n} (X_i - \bar{X})(Y_i - \bar{Y})\,, \tag{2}$$

with n for the sample's size, X_i and Y_i the individual sample points and $\bar{X}$ and $\bar{Y}$ the mean of samples X and Y. As the standard variation is simply the variance squared, the Pearson Correlation Coefficient is then:

$$R_{X,Y} = \frac{\sum_{i=1}^{n} (X_i - \bar{X})(Y_i - \bar{Y})}{\sqrt{\sum_{i=1}^{n} (X_i - \bar{X})^2}\sqrt{\sum_{i=1}^{n} (Y_i - \bar{Y})^2}}\,. \tag{3}$$

The coefficient returns a value between -1 and 1 that represents the limits of correlation from a full negative correlation to a full positive correlation. A value of 0 means no correlation. The value must be interpreted, where often a value below -0.5 or above 0.5 indicates a notable correlation, and values below those values suggests a less notable correlation.

To get the Pearson correlation coefficient for our data, we tested the SciPy function scipy.stats.pearsonr, wich gives us the correct value for each date, and also the np.corrcoef from numpy wich gives the same result.
